# Heat Generated by Different Light-Emitting Diode (LED) Curing Units and Their Effect on Pulp: An In Vitro Study

**DOI:** 10.7759/cureus.88506

**Published:** 2025-07-22

**Authors:** Santhosh Kumar, Jithesh Kumar, Panjami Marish, M Kirthiga, Mora Sathi Rami Reddy, Kuldeep DMello

**Affiliations:** 1 Department of Orthodontics, Adhiparasakthi Dental College and Hospital, Chennai, IND; 2 Department of Orthodontics, Mahe Institute of Dental Sciences and Hospital, Mahe, IND; 3 Department of Conservative Dentistry and Endodontics, Indira Gandhi Institute of Dental Sciences, Sri Balaji Vidyapeeth (Deemed to be University), Puducherry, IND

**Keywords:** histopathologic diagnosis, led curing lights, orthodontic bonding, pulpal inflammation, thermal effects

## Abstract

Introduction: Excessive intensity of light emitted from light-emitting diode (LED) curing units used during orthodontic bonding procedures can induce thermal changes within the pulp chamber, potentially leading to pulpal inflammation or damage. The degree of this thermal effect may vary based on the type and power output of the curing light used. This study aimed to determine the amount of heat generated by different LED curing lights during orthodontic bonding and to evaluate the associated histopathological changes in pulpal tissue.

Materials and methods: The study was conducted on 42 first maxillary premolars scheduled for extraction as part of fixed orthodontic treatment, with informed consent and ethical clearance. The samples were divided into six groups, namely, Group A: Ivoclar Bluephase (histological; Ivoclar Vivadent, Schaan, Liechtenstein), Group B: Woodpecker (histological; Guilin Woodpecker Medical Instrument Co., Ltd., Guilin, China), Group C: 3M Unitek (histological; 3M, Maplewood, MN), Group D: Ivoclar Bluephase (thermal) Group E: Woodpecker (thermal), and Group F: 3M Unitek (thermal). A thermocouple was utilized to assess temperature changes in the pulp chamber before and after the curing process. The histopathological evaluation involved fixing and staining specimens for microscopic examination to identify inflammatory changes. Intergroup analysis of histopathological findings was performed using the chi-square test, while intragroup analysis of pulp chamber temperature changes within the thermal groups was carried out using one-way analysis of variance (ANOVA). A p-value of < 0.05 was considered statistically significant.

Results: No significant histological changes were observed in Groups A and C. Group B exhibited mild to moderate inflammation in six samples, characterized by vascular dilation and inflammatory cell infiltration, with a statistically significant intergroup difference (p = 0.047). Group E showed the highest temperature rise (2.586 ± 0.6744 °C), though the difference among groups was not statistically significant (p = 0.064). One-way ANOVA revealed significant variation in pre-curing (p = 0.001) and post-curing temperatures (p = 0.011), with 3M Unitek showing the highest values in both.

Conclusion: LED curing units with higher light intensity, such as the Woodpecker unit, generated greater heat in the pulp chamber, potentially leading to early inflammatory changes in pulpal tissue. While these changes were predominantly reversible, a sustained temperature rise exceeding 5.5°C may contribute to pulpal irritation.

## Introduction

Light-curing units (LCUs) have become indispensable in contemporary dental practice due to their efficiency, user-friendliness, widespread availability, and potential to reduce chairside time. Traditionally, quartz-tungsten-halogen (QTH) and xenon arc lamps were employed to activate light-curable materials, including those utilized in orthodontic applications. Although cost-effective, these earlier LCUs exhibited limitations, such as a limited operational lifespan, extended curing times, and increased heat generation [1-3].

The advent of light-emitting diode (LED) technology in the late 1990s revolutionized photopolymerization in dentistry [4,5]. Early-generation LEDs were based on gallium nitride (GaN), a semiconductor material capable of producing the blue light required to activate common photoinitiators like camphorquinone. First-generation LED units had irradiance levels below 300 mW/cm² and limited clinical efficacy. The second generation improved on this by delivering irradiance levels approaching 1000 mW/cm², while third-generation "polywave" or "multi-peak" LEDs now exceed 3000 mW/cm² and emit across a broader wavelength range, enabling the activation of multiple photoinitiators [6-8]. In this study, six groups of curing units were selected, comprising different generations and irradiance levels, to compare their thermal effects and assess the associated histopathological changes. This comparison helps determine whether advancements in curing technology compromise pulp safety.

While higher irradiance levels allow for shorter curing times, they also raise concerns about thermal effects on the pulp tissue. During polymerization, heat is generated within the composite resin and transmitted to the underlying dentin and pulp [9]. Dental pulp is a highly vascularized and innervated tissue, and maintaining thermal homeostasis is crucial to prevent adverse biological responses. Elevated intra-pulpal temperatures can disrupt pulpal blood flow, damage odontoblasts, and result in irreversible pulpitis or necrosis. A seminal study illustrated that a temperature rise of merely 5.5°C could lead to irreversible damage in a significant percentage of cases [10].

Although various dental procedures, such as interproximal enamel reduction, polishing, and debonding, can contribute to intra-pulpal thermal stress, this in vitro study specifically aims to assess temperature changes and their histological effects induced by LCUs alone. Reported mechanisms of thermal injury include disruption of dentinal fluid dynamics, vascular coagulation, and direct pulpal necrosis, particularly when intra-pulpal temperatures exceed 42.5°C [11].

The present study aims to evaluate the heat generated by three distinct LED curing units and to assess their potential impact on the dental pulp through both temperature measurements and histological analysis. The primary objective of this in vitro study was to compare the intra-pulpal temperature rise associated with three different high-intensity LED LCUs during orthodontic bonding procedures. The secondary objective was to assess and correlate the histopathological pulpal changes induced by these thermal variations in extracted human premolars. By evaluating both thermal and biological responses, the study aims to provide insights into the potential clinical implications of using different curing technologies in routine orthodontic practice.

## Materials and methods

The study was conducted on patients aged between 15 and 25 years undergoing fixed orthodontic treatment who required the extraction of maxillary first permanent premolars for malocclusion correction. Inclusion criteria comprised non-carious, intact premolars with closed apices and no history of trauma or previous dental procedures. Teeth with developmental anomalies, restorations, caries, or signs of pulpal or periapical pathology were excluded. This age range was selected to minimize variability in pulp chamber size, which may act as a confounding factor in assessing intra-pulpal temperature changes and histological responses. Informed consent was obtained from each patient, and ethical clearance was granted by the Institutional Review Board of Mahe Institute of Dental Sciences and Hospital, Mahe, India, certified under the number MINDS/PG-ETHICAL/15/2020-21.

Sample selection

A total of 42 maxillary premolar samples, all obtained from therapeutically extracted teeth, were included in the study. Of these, 21 premolars (n = 21) were used for evaluating heat generation using thermocouple measurements, while the remaining 21 premolars (n = 21) were allocated for histopathological evaluation. The sample size was calculated using G*Power software (version 3.1.9.7, Heinrich-Heine-Universität Düsseldorf, Düsseldorf, Germany) to achieve a statistical power of 0.80 and a significance level of 0.05. Based on preliminary data indicating a moderate effect size (Cohen’s d = 0.5), the minimum required sample size per group was estimated to be 7 (n = 7).

Experimental groups

The samples were divided into 6 groups: Groups A, B, C, D, E, and F, with n = 7 samples per group. Group A (n = 7): Extracted premolars bonded with brackets and cured using the Ivoclar Bluephase (Ivoclar Vivadent, Schaan, Liechtenstein) curing unit for histopathological evaluation; Group B (n = 7): Extracted premolars bonded with brackets and cured using the Woodpecker curing unit (Guilin Woodpecker Medical Instrument Co., Ltd., Guilin, China) for histopathological evaluation; Group C (n = 7): Extracted premolars bonded with brackets and cured using the 3M Unitek curing unit (3M, Maplewood, MN) for histopathological evaluation; Group D (n = 7): Extracted premolars bonded with brackets and cured using the Ivoclar Bluephase curing unit for heat generation measurement; Group E (n = 7): Extracted premolars bonded with brackets and cured using the Woodpecker curing unit for heat generation measurement; and Group F (n = 7): Extracted premolars bonded with brackets and cured using the 3M Unitek curing unit for heat generation measurement.

Curing unit specifications

**Figure 1:**
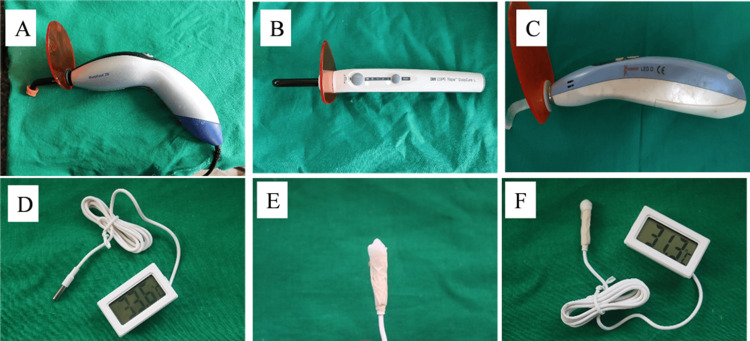
(A) Ivoclar Bluephase curing unit; (B) 3M Unitek curing unit; (C) Woodpecker curing unit; (D) AP Tech digital thermo caliper; (E) thermocouple sensor attached to the tooth retrograde; (F) thermocaliper and tooth assembly

The light intensity (irradiance) of each curing unit was measured in mW/cm² prior to the experimental procedure using a calibrated radiometer (Ivoclar Vivadent). The irradiance values for each unit were Ivoclar Bluephase: 800 mW/cm² (Figure 1A), Woodpecker: 1700 mW/cm² (Figure 1B), and 3M Unitek: 1200 mW/cm² (Figure 1C). These measurements were taken at the focal point of the light tip, which was positioned 1 mm away from the surface of the premolar.

Heat generation measurement

For the heat generation analysis, 21 premolars were allocated and thoroughly cleaned to remove debris and soft tissue. Pulp tissue was carefully extirpated in a retrograde manner through a small apical access cavity created using a round bur under water spray, thereby preserving the crown structure. This access was also used to insert the thermocouple. A digital thermo-caliper (AP Tech, Jipvi Tools Pvt. Ltd., New Delhi, India) integrated with a Type-K thermocouple sensor was used to measure intra-pulpal temperature (Figures 1D, 1E). The thermocouple was calibrated against a reference thermometer (Fluke 1523, Fluke Corporation, Everett, WA) to ensure accuracy.

To maintain consistency in measurement, the thermocouple was positioned 2 mm apical to the pulp chamber roof and stabilized using flowable composite resin, which was light-cured to fix the sensor in place and prevent displacement during curing (Figures 1D, 1F). The correct positioning was confirmed using periodontal probing and visual inspection. All temperature measurements were performed in a controlled laboratory setting with room temperature maintained at 24 ± 1°C and relative humidity at ~50%. Temperature readings were recorded immediately before and after curing.

Standardization of bracket base size and composite thickness

All brackets used were stainless steel premolar brackets with identical base dimensions and mesh design (0.022" slot; 3M Unitek), eliminating variation in base size and geometry. The amount of Transbond XT composite resin (3M) used beneath each bracket was standardized by using a calibrated composite placement instrument to ensure consistent thickness across all samples. The same quantity of resin was used for each sample, and excess was removed uniformly with a dental probe after bracket placement.

Bonding protocol

Enamel surfaces were etched with 37% phosphoric acid for 15 seconds, rinsed for 10 seconds, and air-dried. A thin layer of Transbond XT primer (3M) was applied, and the standardized amount of composite was placed under the bracket, followed by placement and cleanup.

Each sample was light-cured for five seconds using one of the three LED curing units. The curing light was positioned perpendicularly at a fixed distance of 2 mm from the bracket using a custom jig to ensure uniformity. All procedures were conducted by the same operator to avoid inter-operator variability. After curing, the temperature inside the pulp chamber was immediately recorded again, and the rise in temperature was noted for statistical analysis.

The choice of five-second curing time was made to simulate modern high-intensity clinical protocols and maintain consistency, as supported by prior studies (e.g., Maccarini et al. [12]).

Histopathological evaluation

**Figure 2:**
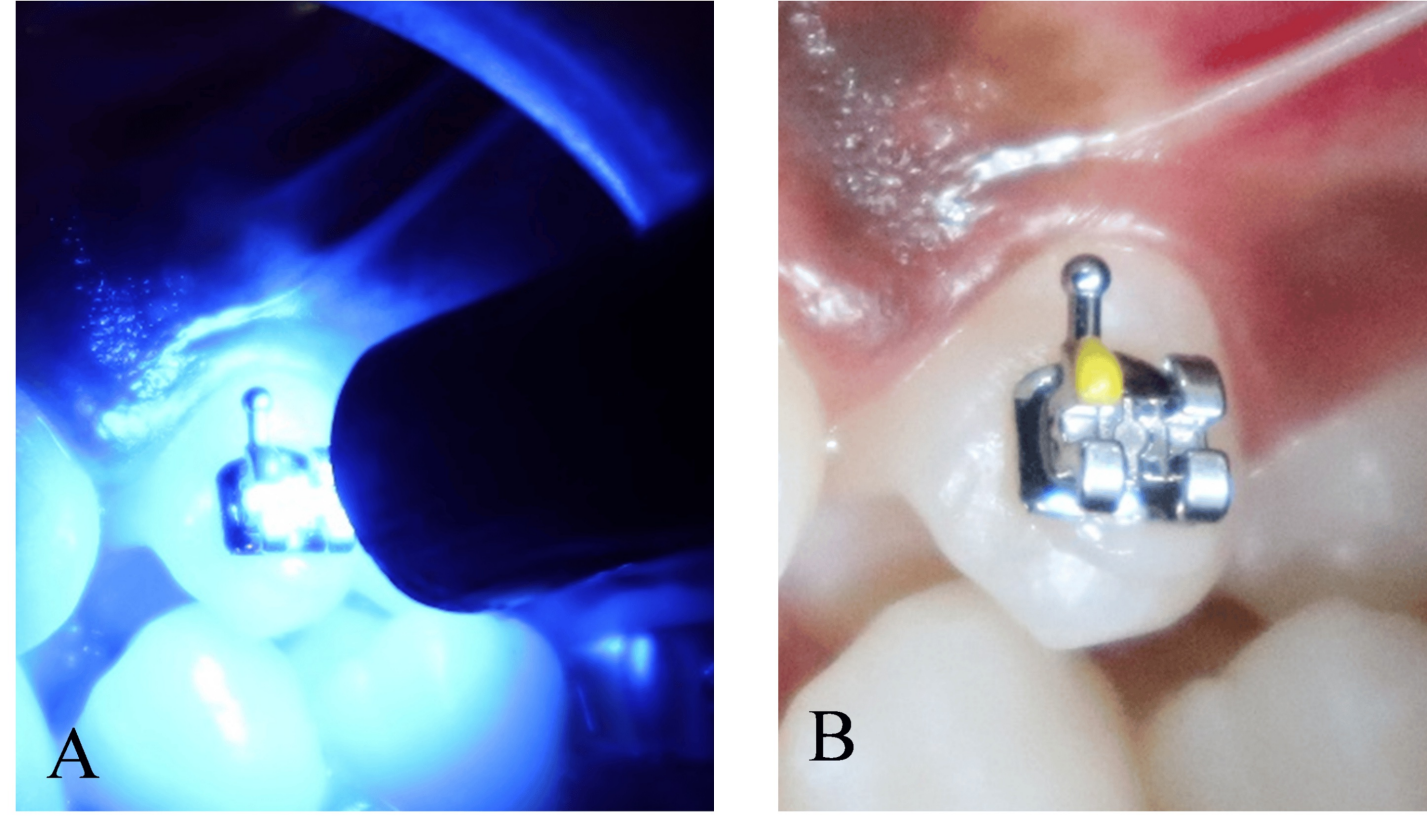
(A) Bonding a bracket with curing light to the tooth surface; (B) bonding a bracket to a tooth intended to be extracted

For histopathological evaluation, the 21 premolars slated for therapeutic extraction were bonded with brackets as described above. The premolars were assigned to one of the three curing light groups (Groups A, B, and C) based on the curing unit used (Figures 2A, 2B).

After the bonding process, the patients were referred to the Department of Oral and Maxillofacial Surgery, and the bonded premolars were extracted within one hour to allow for early detection of thermal-induced histopathological changes. The extracted teeth were carefully cleaned, and a hole was drilled in the apical region of each tooth. The teeth were then immersed in a 10% formaldehyde solution for fixation. Subsequently, the teeth were sent to the Department of Oral Pathology, where they underwent decalcification using 5% nitric acid (HNO₃). After decalcification, the teeth were sectioned using a microtome, and the sections were fixed and stained with hematoxylin and eosin to create slides for microscopic evaluation.

The study incorporated assessor blinding, where the pathologist evaluating the histological slides was unaware of the group allocation. This blinding ensured an objective assessment of inflammatory changes without prior knowledge of the experimental conditions. Inter- and intra-observer reliability were assessed using Cohen’s kappa statistic. Two independent oral pathologists evaluated a randomly selected subset of 10 samples and re-evaluated them after a two-week interval. The inter-observer kappa value was 0.81, and the intra-observer kappa value was 0.85, indicating substantial to almost perfect agreement.

**Figure 3:**
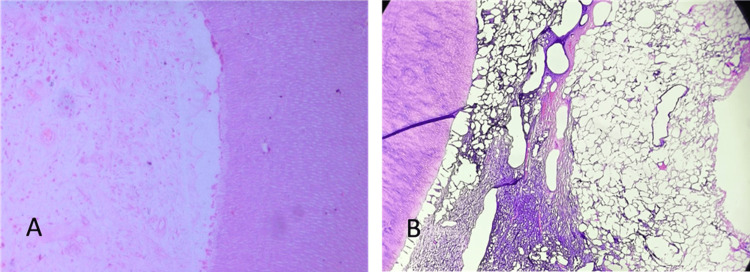
(A) Normal pulp; (B) reversible pulpitis

Under microscopic evaluation, there were no changes in the odontoblast/cell-free/cell-rich or pulp core layer in the pulp. Two samples each from Group A (n = 7) and Group C (n = 7), and six samples from Group B (n = 7), showed dilation of small vessels beneath the coronal pulp, with very few inflammatory cells (mostly plasma cells) identified, suggesting mild to moderate inflammation that can be reversed (Figures 3A, 3B). The resulting data were analyzed, tabulated, and subjected to statistical analysis.

Statistical analysis

The results were statistically analyzed using IBM SPSS Statistics for Windows, version 24.0 (IBM Corp., Armonk, NY). One-way analysis of variance (ANOVA), followed by Tukey’s post hoc test, was used to compare intra-pulpal temperature rise among three different LED curing units. The association between curing unit type and histopathological inflammation was evaluated using the chi-square test.

## Results

The association between LED curing unit type and pulpal inflammation was assessed using the chi-square test (χ² = 6.109, p = 0.047) (Table 1). Group B (Woodpecker) exhibited the highest incidence of inflammation, with six out of seven samples showing mild to moderate changes, such as dilated vessels and scattered inflammatory cells. Groups A (Ivoclar Bluephase) and C (3M Unitek) each showed inflammation in only two out of seven samples, while the remaining samples had no inflammation. The results from Table 1 suggest that the Woodpecker curing unit may be more likely to induce histopathological signs of inflammation compared to Ivoclar and 3M Unitek units. This indicates that the curing unit influences pulpal tissue response.

**Table 1:** Chi-square test for histopathological change

Histopathological report	Ivoclar Bluephase: Group A	Woodpecker: Group B	3M Unitek: Group C	Effect size	Chi-square	P-value
No signs of inflammation	5	1	5	0.54	6.109	0.047*
Signs of inflammation	2	6	2			

In Table 2, a significant difference in pre-curing pulp chamber temperature was observed (ANOVA = 7.138, p = 0.001), with 3M Unitek showing the highest temperature (30.971°C ± 0.8939), followed by Woodpecker (29.429°C ± 0.8655) and Ivoclar (29.071°C ± 0.6102). Post curing, a statistically significant difference in intra-pulpal temperature was observed among the groups (p = 0.011), with the 3M Unitek unit producing the highest mean temperature (32.900 ± 0.7118°C) and the Ivoclar unit the lowest (30.971 ± 0.9995°C). Pairwise comparisons showed a large effect size between 3M Unitek and Ivoclar (Cohen’s d = 2.22) and between Woodpecker and Ivoclar units (d = 1.01), while a moderate effect size was observed between 3M Unitek and Woodpecker (d = 0.65).

**Table 2:** Intergroup comparison between the temperature parameters with the study samples by using an ANOVA test *p-value less than or equal to 0.05 is considered a statistically significant difference.

Parameter	Groups	Mean	SD	95% Confidence interval		Effect size	ANOVA	Significance
				Lower	Upper			
Temperature in the pulp chamber space before curing	Ivoclar Bluephase curing unit	29.071	.6102	28.507	29.636	0.35	7.138	.001*
	Woodpecker curing unit	29.429	.8655	28.628	30.229			
	3M Unitek curing unit	30.971	.8939	30.145	31.798			
Temperature in the pulp chamber space after curing	Ivoclar Bluephase curing unit	30.971	.9995	30.047	31.896	0.33	6.655	.011*
	Woodpecker curing unit	32.186	1.3850	30.905	33.467			
	3M Unitek curing unit	32.900	.7118	32.242	33.558			
Rise in pulp chamber temperature	Ivoclar Bluephase curing unit	1.614	.8688	.811	2.418	0.11	1.720	.064
	Woodpecker curing unit	2.586	.6744	1.962	3.209			
	3M Unitek curing unit	1.929	.6291	1.347	2.510			

The post hoc Tukey's test (Table 3) revealed significant pairwise differences in pre-curing temperatures: 3M Unitek had higher temperatures than Ivoclar (p = 0.001, mean difference = -1.900°C) and Woodpecker (p = 0.005, mean difference = -1.5429°C), while Ivoclar and Woodpecker showed no significant difference (p = 0.687). Post curing, only Ivoclar and 3M Unitek differed significantly (p = 0.009, mean difference = -1.9286°C), with 3M Unitek again higher. No significant differences were found between Ivoclar and Woodpecker (p = 0.112) or between Woodpecker and 3M Unitek (p = 0.440). Regarding the rise in temperature, no pairwise comparison was significant, though Ivoclar and Woodpecker approached significance (p = 0.057), suggesting a trend for Woodpecker inducing a higher rise. In conclusion, 3M Unitek consistently showed higher temperatures, but the rise in temperature was similar across the units, indicating that the higher post-curing temperatures with 3M Unitek are due to its higher baseline rather than greater heat generation. 

**Table 3:** Post hoc analysis test using Tukey's test *The mean difference is significant at the 0.05 level.

Parameter	Groups	Comparison groups	Mean difference	Significance	95% Confidence interval		Effect size Cohen’s d
					Lower	Upper	
Temperature in the pulp chamber space before curing	Ivoclar Bluephase curing unit	Woodpecker curing unit	-.3571	.687	-1.449	.734	0.48
		3M Unitek curing unit	-1.9000^*^	.001	-2.991	-.809	2.48
	Woodpecker curing unit	Ivoclar blue phase curing unit	.3571	.687	-.734	1.449	0.48
		3M Unitek curing unit	-1.5429^*^	.005	-2.634	-.451	1.75
	3M Unitek curing unit	Ivoclar blue phase curing unit	1.9000^*^	.001	.809	2.991	2.48
		Woodpecker curing unit	1.5429^*^	.005	.451	2.634	1.75
Temperature in the pulp chamber space after curing	Ivoclar Bluephase curing unit	Woodpecker curing unit	-1.2143	.112	-2.672	.243	1.01
		3M Unitek curing unit	-1.9286^*^	.009	-3.386	-.471	2.22
	Woodpecker curing unit	Ivoclar blue phase curing unit	1.2143	.112	-.243	2.672	1.01
		3M Unitek curing unit	-.7143	.440	-2.172	.743	0.65
	3M Unitek curing unit	Ivoclar blue phase curing unit	1.9286^*^	.009	.471	3.386	2.22
		Woodpecker curing unit	.7143	.440	-.743	2.172	0.65
Rise in pulp chamber temperature	Ivoclar Bluephase curing unit	Woodpecker curing unit	-.9714	.057	-1.969	.026	1.25
		3M Unitek curing unit	-.3143	.705	-1.312	.684	0.41
	Woodpecker curing unit	Ivoclar blue phase curing unit	.9714	.057	-.026	1.969	1.25
		3M Unitek curing unit	.6571	.240	-.341	1.655	1.01
	3M Unitek curing unit	Ivoclar blue phase curing unit	.3143	.705	-.684	1.312	0.41
		Woodpecker curing unit	-.6571	.240	-1.655	.341	1.01

## Discussion

Numerous studies have investigated the thermal behavior of LCUs in restorative dentistry. Bagis et al. have reported that LED curing lights typically generate less heat compared to traditional halogen units, attributable to their enhanced energy efficiency and specific spectral targeting [13]. However, subsequent research has highlighted that light intensity, or irradiance, is a more critical factor in determining the temperature rise within the pulpal chamber than the type of curing unit employed.

Oberholzer et al. demonstrated a direct correlation between increasing irradiance and the elevation of intra-pulpal temperature across various curing systems [14]. Armellin et al. reinforced this finding in their study comparing two LED units operating at irradiance levels of 1000 mW/cm² and 3200 mW/cm², respectively, observing significantly elevated temperatures within the pulp chamber at the higher intensity. Similarly, Hannig and Bott concluded that light intensity, rather than the type of light source, is the principal factor affecting pulpal heat generation [15]. The LED curing lights used in the present study emitted wavelengths near 470 nm, optimal for activating camphorquinone, the most common photoinitiator in dental composites. Units with higher intensities (>2000 mW/cm²) are marketed to reduce curing time and improve polymerization [16]. However, this benefit must be weighed against their potential to induce intra-pulpal temperature increases exceeding 5.5°C, a threshold that Zach and Cohen famously associated with irreversible pulpal damage in 15% of cases [17].

Previous research has shown that LED LCUs exhibit favorable performance for achieving sufficient depth of cure, flexural strength, and surface microhardness [18]. Dental pulp can perform its vital function within a particular temperature range. An abnormal rise in pulp chamber temperature can cause inevitable damage to pulpal tissue [19].

Recent studies continue to support the association between LED curing light intensity and pulp chamber temperature rise. Maucoski et al. (2023) demonstrated that high-power LED units such as the Quadwave produce a significantly greater intra-pulpal temperature increase compared to conventional units, emphasizing the clinical relevance of output intensity and exposure time [20]. Similarly, Mouhat et al. (2021) highlighted that thermocouple sensors remain a reliable method for detecting subtle pulp temperature variations in both in vitro and in vivo conditions [21]. These findings align with earlier work by Armellin et al., who reported that curing lights with higher irradiance levels (3200 mW/cm²) led to more pronounced temperature increases compared to those with lower outputs (1000 mW/cm²) [22].

In our study, the maximum temperature rise observed was approximately 2.7°C, which is below the critical threshold of 5.5°C reported to cause irreversible pulpal damage. However, even sub-threshold thermal elevations can result in mild, reversible pulpal inflammation, especially when additional thermal stimuli are present or when dentin thickness is reduced, limiting thermal buffering. It is critical to note that in vivo, pulpal blood flow acts as a natural protective mechanism by dissipating heat, thereby reducing the risk of thermal injury. As such, direct extrapolation from in vitro results may overestimate the actual risk of pulpal inflammation in clinical conditions.

Notably, the Woodpecker LED unit (1700 mW/cm²) caused the greatest temperature rise and the most frequent inflammatory changes. Histologically, six out of seven samples (85.7%) exposed to this unit exhibited signs of inflammation, including two with moderate severity. This can be attributed to both the higher output power and potentially tighter beam collimation, which increases energy concentration and heat delivery to the pulp chamber. In contrast, the Ivoclar Bluephase (800 mW/cm²) and 3M Unitek (1200 mW/cm²) groups showed only two mild cases of inflammation each, with negligible temperature rise. These findings suggest a dose-dependent biological response to irradiance level. Although the inflammatory changes observed were mild and likely reversible, they raise concern when high-intensity curing units are used in deep cavities or on young teeth with large pulp chambers.

A key limitation of this study is that it was conducted on extracted human premolars under in vitro conditions, which do not replicate the dynamic biological environment present in vivo. In clinical scenarios, pulpal blood flow serves as a thermal sink, dissipating heat and potentially mitigating the inflammatory response to thermal stimuli. As a result, the histopathological findings observed in this study may overestimate the actual severity of pulpal inflammation induced by light-curing in a clinical setting.

## Conclusions

The study findings indicate that among the three tested LED curing units, the Woodpecker light produced the highest intra-pulpal temperature rise, followed by Ivoclar Bluephase and 3M Unitek. Histological evaluation revealed mild inflammatory changes in some samples, particularly in groups exposed to higher temperatures. Although the observed temperature rise remained below the critical threshold for irreversible pulp damage (5.5°C), subclinical pulpal responses were evident. These results suggest that even short-duration exposure to high-intensity LED curing units can induce mild biological effects on pulp tissue. However, given the in vitro design, small sample size, and lack of standardized energy output across units, these findings should be interpreted with caution and warrant further clinical investigation.
